# Monitoring treatment in pediatric patients with 21-hydroxylase deficiency

**DOI:** 10.3389/fendo.2023.1102741

**Published:** 2023-02-03

**Authors:** Tomoyo Itonaga, Yukihiro Hasegawa

**Affiliations:** ^1^ Department of Pediatrics, Oita University Faculty of Medicine, Oita, Japan; ^2^ Division of Endocrinology and Metabolism, Tokyo Metropolitan Children’s Medical Center, Tokyo, Japan; ^3^ Department of Pediatrics, Keio University School of Medicine, Tokyo, Japan

**Keywords:** urine pregnanetriol, 17-hydroxyprogesterone, 21-hydroxylase deficiency, congenital adrenal hyperplasia, first morning urine sample

## Abstract

21-hydroxylase deficiency (21-OHD) is the most common form of congenital adrenal hyperplasia. In most developed countries, newborn screening enables diagnosis of 21-OHD in asymptomatic patients during the neonatal period. In addition, recent advances in genetic testing have facilitated diagnosing 21-OHD, particularly in patients with equivocal clinical information. On the other hand, many challenges related to treatment remain. The goals of glucocorticoid therapy for childhood 21-OHD are to maintain growth and maturation as in healthy children by compensating for cortisol deficiency and suppressing excess adrenal androgen production. It is not easy to calibrate the glucocorticoid dosage accurately for patients with 21-OHD. Auxological data, such as height, body weight, and bone age, are considered the gold standard for monitoring of 21-OHD, particularly in prepuberty. However, these data require months to a year to evaluate. Theoretically, biochemical monitoring using steroid metabolites allows a much shorter monitoring period (hours to days). However, there are many unsolved problems in the clinical setting. For example, many steroid metabolites are affected by the circadian rhythm and timing of medication. There is still a paucity of evidence for the utility of biochemical monitoring. In the present review, we have attempted to clarify the knowns and unknowns about treatment parameters in 21-OHD during childhood.

## Introduction

**Figure 1:**
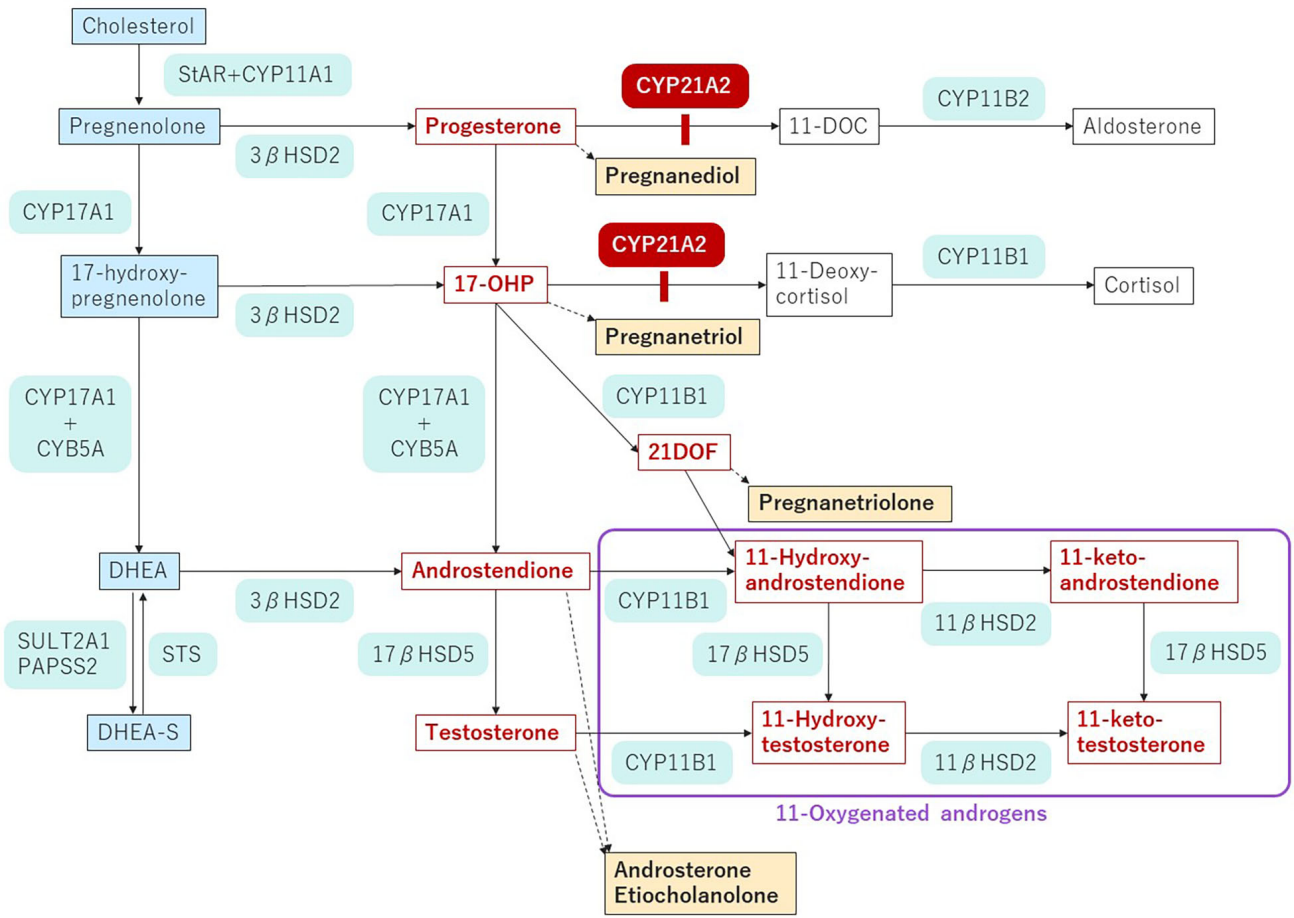
Pathway of steroidogenesis in 21-OHD.The enzyme blockade in 21-OHD is indicated by the red bar. Androgens and androgen precursors are indicated in red font. Urinary metabolites are indicated by yellow highlighting. StAR, steroidogenic acute regulatory protein; CYP, cholesterol side chain cleavage; HSD, hydroxysteroid dehydrogenase; CYB, cytochrome; DOC, deoxycorticosterone; 17-OHP, 17-hydroxyprogesterone; 21-DOF, 21-Deoxycortisol; DHEA, dehydroepiandrosterone; DHEA-S, dehydroepiandrosterone sulfate; STS, steroid sulfatase; SULT, sulfotransferase; PAPSS2, 3’-phospho-5’-adenylyl sulfate.

21-hydroxylase deficiency (21-OHD), the most common form of congenital adrenal hyperplasia, is an autosomal recessive disease caused by mutations in *CYP21A2* and has an incidence of 1:15,000-18,000 births (1, 2). *CYP21A2* encodes adrenal steroid 21-hydroxylase (P450c21), an enzyme which converts 17-hydroxyprogesterone (17OHP) to 11-deoxycortisol and progesterone to deoxycorticosterone, the precursors of cortisol and aldosterone, respectively. The blockade of cortisol synthesis leads to corticotropin stimulation of the adrenal cortex; the resulting accumulation of the precursors is diverted to sex hormone biosynthesis (**Figure 1**).

Newborn screening, now performed in many developed countries, leads to early diagnosis of 21-OHD, especially of the classical phenotype. Moreover, recent advances in genetic testing have solved certain problems related to diagnosing 21-OHD. On the other hand, many challenges related to treatment remain. Managing patients with 21-OHD using glucocorticoids (GCs) is not optimal, and additional treatments have been proposed (3–5), such as the androgen antagonist, flutamide; the aromatase inhibitor, testolactone; the P450c17 inhibitor, abiraterone acetate (6, 7); the corticotropin-releasing factors, receptor 1 antagonist crinecerfont (8, 9) and tildacerfont (10); ACTH antagonists (11, 12); and melanocortin type 2 receptor antagonist (13, 14).

Indices for managing mineralocorticoid (MC) treatment include blood pressure and serum electrolyte and plasma renin levels (3, 4). However, the optimal dosage for MC substitution has not been critically studied. Sensitivity to MC is relatively lower during the neonatal and early infantile periods than later in life owing to immature tubular reabsorption of sodium, making MC treatment difficult. There are also unresolved challenges concerning the monitoring of MC treatment.

In the present review, we addressed the following topics, focusing on challenges related to monitoring the GC treatment for 21-OHD during childhood.

1) Goals of 21-OHD treatment in childhood
2) Gold standard of monitoring pediatric 21-OHD
3) Blood sampling to monitor 21-OHD treated with GC
4) Utility of urine sampling to monitor 21-OHD
5) Future prospects

## Goals of 21-OHD treatment in childhood

The goals of treating childhood 21-OHD are preventing adrenal crisis and virilization and maintaining growth and maturation comparable to those of healthy children (3, 4). Under-treatment causes adrenal insufficiency, such as weight loss, anorexia, gastrointestinal complaints, weakness, and fatigue. Insufficient cortisol synthesis in patients with 21-OHD also leads to an impaired negative feedback drive to the hypothalamus and pituitary or increased ACTH secretion, resulting in excess 17OHP and adrenal androgens. Starting in the fetal period, excess androgens induce masculinization in female patients. Abnormally increased height velocity and acceleration of bone maturation occur in both sexes. The abnormal advancement of bone maturation results in loss of growth potential and short stature. In this regard, GC therapy, which mainly consists of hydrocortisone (HDC) for children with 21-OHD, aims to compensate for the cortisol deficiency and suppress excess adrenal androgen production (3, 4). However, it is not easy to calibrate the GC dosage accurately in patients with 21-OHD. Even if physiological HDC can be supplemented to counteract the cortisol deficiency, it cannot suppress ACTH secretion completely. Therefore, HDC requirements exceed the physiological dosage whereas excessive treatment causes iatrogenic Cushing’s syndrome characterized by short stature, obesity, and hypertension. Taken together, both under- and over-treatment can lead to a lower quality of life.

Patients with 21-OHD often attain final height (FH) that is significantly lower than their parentally determined, target height. In a 2001 meta-analysis of final height based in 18 studies conducted between 1977 and 1998 (15), the mean final height SD score (FH SDS) was -1.57 for male patients and -1.24 for female patients for an average of -1.37. A second meta-analysis of 35 studies conducted between 1966 and 2007 showed an average FH SDS of -1.38 and a corrected FH SDS of -1.03 (16). A recent study reported mean FH SDS values of -0.4 to -1.4 (17–23). Good compliance, early diagnosis (age <1 year), and short duration of HDC treatment in the peripubertal period reportedly improved FH (15, 19, 20, 22). For example, FH positively correlated with height at age 2 years in both sexes (24). It also positively correlated with the age at menarche in female salt-wasting (SW) and simple virilizing (SV) groups (24). Furthermore, the major determinant of FH differed among the various phenotypes of 21-OHD (24). Other critical factors determining FH are age at puberty initiation and pubertal height gain (25–29). In addition, most studies of FH revealed a sex difference; FH SDS was higher in female patients than in male patients (15, 19–22). This difference was thought to stem from the tendency toward a later diagnosis in male patients with the SV form.

Achieving normal pubertal development is another important goal for 21-OHD patients, in whom the onset of puberty varies (17, 24, 30). Puberty began significantly earlier in female patients with 21-OHD with the SW and SV forms than in those with the non-classic forms or the general female population whereas puberty in male patients began earlier only in patients with the SV form (24). In another study enrolling more subjects, patients of both sexes had earlier than normal onset of puberty (30). On the other hand, yet another study reported that breast development or menarche did not occur earlier in female patients with 21-OHD (17). These studies presumably referred to central puberty; however, it should be noted that some of the studies failed to describe the inclusion criteria for androgen excess.

Pubertal height gains also differed among previous reports. One reason for this difference is that the definition of pubertal height gain varied among studies. Nonetheless, whatever the definition, several studies reported less pubertal height gain in patients with 21-OHD than in a population of healthy subjects (17, 24–29). The latest study, in which all the subjects received a diagnosis of 21-OHD in the neonatal period, demonstrated that the pubertal growth spurt was similar to that of healthy children of both sexes (23). This apparently normal pubertal development suggests that early diagnosis *via* newborn screening enabled normalization of pubertal height gain and FH. However, no studies have as of yet compared differences in pubertal height gain and FH before and after the introduction of newborn screening.

In adolescents, irregular menstruation and testicular adrenal rest tumors (TART), which are associated with poorly controlled disease, are also of clinical concern. Female patients in whom 21-OHD is well controlled usually experience normal menarche (30). Therefore, irregular menstruation rarely persists in this condition. On the other hand, the prevalence of TART reportedly increases to 20-30% from ages 10 to 18 years (31–33). Regular testicular ultrasonogram screening for TART is recommended every year to two years in male patients aged 10 years or older (3, 4). It remains to be determined whether episodes of poor disease control can influence the development of TART and later gonadal function.

## Gold standard of monitoring pediatric 21-OHD

Calibrating medication dosages for 21-OHD is difficult (3, 4, 34–37). The appropriate dosage for maintenance therapy differs among individuals for various reasons, including differences in disease severity. Auxological data, such as height, body weight, and bone age, are considered the gold standards for monitoring 21-OHD especially during prepuberty (3, 4, 25, 38, 39). The importance of height has already been mentioned above; height can be accelerated or decelerated depending on the HDC dosage. Similarly, weight can be increased by overdosing with HDC while it may be decreased or maintained at an appropriate level with lower dosages. Children older than age 1.5 years should be monitored auxologically every four months or more frequently after any change in dosing (3, 4).

Bone age can also be used as a marker. Generally, bone age can be advanced or retarded in the course of undertreatment or overtreatment of HDC, respectively. At least two points pertinent to HDC use need to be kept in mind: first, bone age should be assessed after age 2 years because even in untreated patients with classic 21-OHD, bone age does not advance until age 1-1.5 years (40). Second, during puberty bone age alone cannot be used as a clinical marker because it cannot accurately predict the FH (23). Generally, a once yearly bone age evaluation is considered sufficient, but evaluations should be performed twice yearly if the growth rate shows a rapid change, or the patient enters puberty (4).

Besides auxological data, clinicians should be alert to the physical signs of skin and mucosal hyperpigmentation and virilization (onset of pubic hair growth, apocrine odor). In addition, signs of GnRH-dependent precocious puberty, such as breast development or testicular enlargement, should be monitored because elevated adrenal androgens may activate the hypothalamic-pituitary-gonadal axis (3).

## Blood sampling to monitor 21-OHD treated with GC

A disadvantage of using auxological data is that the data require months to a year to evaluate. Therefore, a method of monitoring for a shorter period is needed.

Serum 17OHP and androstenedione are traditional indicators of adequate GC treatment for 21-OHD (3, 4). Their values can be obtained after a much shorter monitoring period (hours to days) than is the case with auxological data. ACTH values are not helpful because of fluctuations due to the circadian rhythm (**Table 1**). The utility of monitoring the value of androgens, such as testosterone and DHEA-S, has not been sufficiently studied. Some recent studies have found that metabolites, such as 21-deoxycortisol and 11-oxygenated androgens, may reflect adrenal androgen precursor production (41–43). Liquid chromatography-tandem mass spectrometry (LC-MS/MS) allows us to measure several adrenal androgen precursors simultaneously but is not yet widely available (**Table 1**). The latest international guidelines do not provide specific target levels for steroid measurement because laboratory reference ranges and timing of sample collection vary and the whole clinical picture must be considered (3). However, the target values provided by some studies may be of use to clinicians.

**Table 1:** Pros and Cons of each biochemical measurements.

Sample	Measurement	Pros	Cons
Serum	Common points in the items below	1) Inexpensive 2) Established in clinical care 3) No age limit; available for infants	1) Reflects a shorter period than a urine sample 2) Diurnal fluctuations due to the circadian rhythm and medication
	17OHP by immunoassay (17OHP by LC-MS/MS is still at the research stage)	1) Target range of early morning 17OHP value reported 2) Recommended in guidelines	1) No studies based on auxological data 2) Difficulties in early morning serum sampling
	Androstenedione by immunoassay	1) Recommended in guidelines	1) Target range not based on auxological data 2) Not available in some countries
	ACTH by immunoassay		1) Variability due to circadian rhythm and stress caused by puncture 2) Not recommended in guidelines
DBS	17OHP by LC-MS/MS	1) Ease of sampling in early morning 2) Feasibility in remote medical care	1) DBS-specific reference ranges have not been established 2) Not recommended in guidelines
Urine	Common points in the items listed below	1) Non-invasive 2) Reflects a longer period than a serum sample; overnight in the first morning sample and daily in the 24-hour sample 3) Accurate and standardized	1) Difficulty with obtaining samples from infants
	Pregnanetriol by GC-MS	1) Target range based on prepubertal auxological data	1) Requirement for time-consuming derivatization steps
	GC-MS steroid metabolome analysis	1) Target range based on prepubertal auxological data	1) Not available in some countries 2) Requirement of time-consuming derivatization steps
	LC-MS/MS steroid metabolome analysis	1) No time-consuming derivatization steps	1) Expensive 2) Not yet established in clinical care

ACTH and cortisol secretions fluctuate with the circadian rhythm, with levels normally increasing in the early morning (34, 44). The timing of GC administration can also influence their values (45). Serum 17OHP also follows the rhythm and is similarly affected by medication. Therefore, it is preferable to measure serum 17OHP regularly before administering GC early in the morning (3, 4). The target range of serum 17OHP immediately before the morning GC administration is reportedly 4-12 ng/mL in both children and adults (44) and <5.9 ng/mL during puberty (18). However, these target ranges were arbitrarily determined rather than being based on auxological data (34, 44). The latest study of the topic demonstrated a 17OHP cut-off value >4.3 ng/ml with a sensitivity of 85.48% and specificity of 37.59% in patients younger than 18 years with poorly controlled disease, as defined by an accelerated growth rate (46). It is noteworthy that most 17OHP values used as a clinical marker were obtained by immunoassay. A disadvantage of monitoring using the 17OHP value is the difficulty of sampling blood in hospitals in the early morning, which is outside the regular staff working hours (**Table 1**). To resolve this difficulty, several studies have examined dried blood spot (DBS) samples, especially those recently used with LC-MS/MS (47–50), for their utility for monitoring purposes. However, all these studies were small-scale. Moreover, the use of DBS presents several problems. First, DBS analysis cannot simply replace serum analysis until its robustness has been documented in larger comparative studies and DBS-specific reference ranges have been established. Second, DBS sampling may be difficult to do at home for non-medically trained individuals, especially young pediatric patients (51).

Standardizing 17OHP values is crucial. Currently, two methods of measuring 17OHP exist, namely, the immunoassay and mass spectrometry. However, these methods are not without their challenges. Cross-reactivity with other steroid compounds, especially in neonates, may affect the results of an immunoassay. Further, each assay uses a different antibody, leading to variability in the results (**Table 1**). On the other hand, LC-MS/MS enables accurate measurement of the absolute value of 17OHP and other steroid metabolites (52–54). The optimal therapeutic target range of 4-12 ng/mL for morning serum 17OHP described above (3, 4, 34, 44) was obtained using older, radioimmune assays. It is still uncertain whether the 17OHP values obtained in this way correlate with those obtained with LC-MS/MS (14, 51). Thus, standardization of immunoassay results is needed for LC-MS/MS to become more widely accepted as a measurement method.

## Utility of urine sampling to monitor 21-OHD

In clinical practice, urine sampling is more feasible, is easily done periodically, and has the added advantage of being minimally invasive. Moreover, urine samples may better reflect longer-term conditions than blood samples (**Table 1**). At least three studies using urine samples investigated the optimal monitoring based on auxological data (55–57).

### Pregnanetriol

Pregnanetriol (PT) is a urinary metabolite of 17OHP. Measuring PT by gas chromatography-mass spectrometry (GC-MS) has been proposed as a form of monitoring treatment of 21-OHD (55, 56, 58). Our previous studies, based on the auxological data of height velocity, body weight, and bone age, demonstrated that the morning urine PT value can be used as an index of control in prepubertal patients with 21-OHD (55, 56). In these studies, the criteria for good disease control included maximal changes in the height SDS < ± 0.2/year during the prepubertal period (Tanner stage 1) and changes in body weight SDS < 0.5/year during late puberty. Based on the criteria, the PT level during periods of good disease control ranged from 1.2 to 2.1 mg/m^2^/day (95% confidence interval [CI] for the mean) in 24-hour urine samples (55) and 2.2 to 3.3 mg/gCr (95%CI) and 0.59 to 6.0 mg/gCr (the 10th - 90th percentile) in the first morning urine (56). In conclusion, these ranges could be used as an index of optimal control.

In a recent, prospective study, the first morning PT levels before morning medication significantly correlated with the blood 17OHP value obtained from DBS on filter paper (59). Early morning urine collection, which provides urine data from late night to early morning, is more suitable for repetitive measurements than 24-hour urine collection (**Table 1**).

### Urinary steroid profile by GC-MS

Two studies reported a correlation between blood 17OHP and urinary steroid metabolites on GC-MS analysis for 24 hours (45, 58). Urinary steroid metabolites, including the PT/tetrahydrocortisone ratio, the sum of three 17-hydroxyprogesterone metabolites/the sum of three cortisol/cortisone metabolites ratio, 5α-pregnane-3α, 17α-diol-20-one (a backdoor pathway metabolite), significantly correlated with the early morning 17OHP value.

Most recently, the use of growth rate was recommended as a basis for determining the target value of urinary steroid metabolite excretion in prepubertal children with 21-OHD (57). The target range for the androgen metabolite z-score and the hydrocortisone metabolite, tetrahydrocortisol, was 0.164-0.512 and <1,480 µg/m^2^ body surface area/day, respectively. The study also reported the utility of metabotyping in urinary steroid metabolome analysis using 24-hour urine samples (60). This metabolome analysis recognizes four, unique metabotypes, each with a specific signature, characterized by differences in the cortisol metabolite and androgen metabolite values. Thus, this method may enable the classification of patients into an appropriate, overtreated, undertreated or treatment failure group. However, measuring GC-MS is not feasible at all institutions or in all countries (**Table 1**).

### Urinary steroid profile by liquid chromatography-tandem mass spectrometry

GC-MS analysis is the mainstay of urinary multisteroid profiling (3). Recently, Pussard et al. developed a novel, more extensive LC-MS/MS method for measuring the urinary value of 23 steroids, which demonstrated a close correlation between morning plasma and urinary 17OHP (61). Patients with well-controlled disease had a plasma concentration of 17OHP below 20 ng/mL and a normal androstenedione level before morning medication, indicating lower levels of steroid upstream of the 21-hydroxylase defect and a lower level of androgens (androstenedione, testosterone, and the sum etiocholanolone + androsterone). It is noteworthy that the study used morning spot urine samples as in a previous study of PT (56). If established, this can be a good monitoring method because morning spot urine sampling is feasible and suitable for periodical monitoring. LC-MS/MS method is promising because it is not as time-consuming as GC-MS. Its main drawback is its limited availability (**Table 1**).

## Future prospects

Optimizing 21-OHD treatment in children requires establishing a monitoring method that combines auxological data with biochemical measurements. Ideally, the optimal range of each biochemical indicator would be based on auxological data. Prospective studies of biochemical monitoring based on auxological data can further validate the retrospective results reported thus far.

Little is known about treatment parameters during puberty. Although the latest guidelines do not mention the management of 21-OHD during puberty (3, 4), two, retrospective studies discussing the optimal treatment method for pubertal patients (17, 62) suggested that the pubertal, growth-suppressing effects of HDC outweigh the negative effects of elevated androgens, and that the HDC dosage during puberty should not exceed 17 mg/m^2^/day to optimize FH. However, other studies have not focused on biochemical monitoring during puberty (23–29). It may be difficult to conduct a prospective study of this matter because of the heterogeneity in pubertal progression. Research on monitoring the disease in the neonatal period and infancy faces similar challenges.

Finally, it is unknown how the results of monitoring in the neonatal period, childhood, and adolescence indicating disease control in each period may eventually affect the disease status in adulthood, including complications and quality of life. For example, it remains to be seen how quality of life and complications in patients with 21-OHD improve in adulthood if the condition is diagnosed at an early stage, such as *via* neonatal screening, to enable early intervention. Does poor biochemical control in childhood and adolescence relate to infertility in adulthood? To this and other questions, patients with 21-OHD need to be followed-up continuously. In this sense, a registry system is essential for future research. A high-quality registry system can provide clues to better monitoring methods. Several countries have already established their own, whole case registry system and produced meaningful research outcomes (23, 63–65). If the challenges of monitoring described above can be solved, treatments can be refined to enable patients with 21-OHD to receive appropriate management over the course of their whole life.

## Author contributions

TI and YH contributed substantially to the conception and design of the study, drafting of the manuscript, and its review; have given their final approval of the version submitted; and agree to be accountable for the accuracy and integrity of its content. All authors contributed to the article and approved the submitted version.
